# The Liver-Protective Effects of the Essential Oil from *Amomum villosum* in Tilapia (*Oreochromis niloticus*): Antioxidant, Transcriptomic, and Metabolomic Modulations

**DOI:** 10.3390/antiox13091118

**Published:** 2024-09-16

**Authors:** Hongbiao Dong, Xiangbing Zeng, Xiaoting Zheng, Chenghui Li, Junchao Ming, Jiasong Zhang

**Affiliations:** 1 South China Sea Fisheries Research Institute, Chinese Academy of Fishery Sciences, Key Laboratory of South China Sea Fishery Resources Exploitation & Utilization, Ministry of Agriculture and Rural Affairs, Guangzhou 510300, China; donghongbiao@163.com (H.D.); xiangbing1998@163.com (X.Z.); xtzheng1990@163.com (X.Z.); lichenghui1998@126.com (C.L.); mjc0312@126.com (J.M.); 2 Key Laboratory of Efficient Utilization and Processing of Marine Fishery Resources of Hainan Province, Lingshui 572426, China; 3 Key Laboratory of Mariculture, Ministry of Education, Ocean University of China, Qingdao 266003, China; 4 National Aquaculture Engineering Technology Research Center, Zhejiang Ocean University, Zhoushan 316000, China

**Keywords:** tilapia, essential oil, liver protection, transcriptomics, metabolomics, diet study

## Abstract

This study investigates the effects of the essential oil from *Amomum villosum* (EOA) on liver-protective effects in Nile tilapia (*Oreochromis niloticus*), utilizing a multidisciplinary approach that integrates physiological assessments and transcriptomic and metabolomic analyses. Fish were fed diets containing 2 g/kg of EOA over a 56-day trial, with a no-EOA diet serving as the control. The results demonstrate that EOA supplementation improves liver histology, enhances antioxidant capacities, and reduces inflammation in tilapia. The transcriptomic analysis revealed significant alterations in gene expression profiles related to RNA splicing, metabolism, and disease pathways. The identification of differential genes and disease databases identified key target genes associated with the primary component of EOA for its anti-hepatobiliary disease effects. Furthermore, a molecular docking analysis of EOA major components with core differentially expressed genes in the hepatobiliary syndrome indicated that α-pinene is a potential Hsp90 inhibitor, which may prevent inflammation. A metabolomic analysis further demonstrated that EOA supplementation leads to notable changes in liver phospholipids, fatty acids, and carbohydrate metabolism. These findings underscore the potential of EOA as a natural additive for improving liver health in tilapia, offering valuable insights to the aquaculture industry for enhancing fish health and welfare in intensive farming systems.

## 1. Introduction

As global population levels rise, the great demand for aquatic products has seen a corresponding increase [1]. In China, the aquaculture industry has transitioned to large-scale and intensive models of development to satisfy the need for high-quality aquatic protein [2]. However, this shift has led to significant challenges, including environmental degradation, disease outbreaks, and persistent medication residues [2]. Given the essential role of fish liver in detoxification and digestion, hepatobiliary diseases, encompassing a wide range of liver and biliary system conditions caused by infections, neoplasia, and toxic exposure have become more prevalent [3,4,5].

Nile tilapia (*Oreochromis niloticus*), native to Africa, boasts attributes highly conducive to aquaculture, such as resilience, high reproductive rates, an omnivorous diet, rapid growth, and the absence of intermuscular spines [6]. These traits have not only earned it the nickname “aquatic chicken” but also the endorsement of the Food and Agriculture Organization of the United Nations as an exemplary species for global aquaculture [7,8]. As the world’s leading tilapia producer, China contributes significantly to the global supply, making up 20% of the total yield [6]. The drive towards cost-effective, high-volume production has led to the extended use of plant-based proteins, which has been linked to digestive diseases among tilapia, highlighting the need for improved liver health management [9,10].

Traditional Chinese herbal medicine, known for its safety and efficacy, has gained attention in aquaculture research. These herbal preparations offer multiple benefits, including no drug residues, minimal side effects, cost-effectiveness, and the lack of antimicrobial resistance, improving feed efficiency and aquatic animal health. *Amomum villosum*, or Sharen, a staple in Chinese herbal medicine, has been used for centuries to treat gastrointestinal issues in humans [11,12,13,14]. Its primary bioactive component, the essential oil from *A. villosum* (EOA), has demonstrated potential in protecting the gastrointestinal tract. In another study, we explored dietary EOA’s effects on juvenile tilapia intestines. Our findings reveal that EOA supplementation improved tilapia’s growth and digestive and antioxidant capacity in tilapia [15]. Considering the holistic approach to treating liver and intestinal conditions, this study explores the effects and mechanisms of EOA on tilapia liver disease through an integrated approach of physiology, transcriptomics, and metabolomics, offering valuable insights for enhancing tilapia health cultures.

## 2. Materials and Methods

### 2.1. Experimental Design

Tilapia fingerlings were provided by Baolu Co., Ltd., Haikou, China, and belong to the all-male GIFT Nile tilapia strain. They were reared indoors at the Guangdong Nanwan Co., Ltd., Yangjiang, China, until they reached the experimental size (20.34 ± 2.08 g). These fish were subsequently transported to our experimental tanks for a 14-day acclimation period. The water temperature was consistently set at 26 ± 2 °C, and a pH level was sustained at 7.8 ± 0.1. The natural photoperiod was used, and the water quality indicators were checked daily to maintain them within the safety range. During acclimation, the fish were fed a commercial diet from Haida Feed Co., Ltd., Jiangmen, China, comprising soybean meal, rapeseed meal, and soybean oil. EOA was obtained from Baishengyuan Co., Ltd., Yangjiang, China. It was extracted by the distillation of the stems of *A. villosum*. The main components of EOA are described in Table S2. Based on our previous research, 2 g/kg of EOA is the optimal supplementation level [15]. Therefore, following a 14-day acclimation period, the experiment included two groups: a control group (CON) and a 2 g/kg-of-EOA group (EOA). Each group consisted of four replicates with 30 fish. After weighing, the EOA was added to distilled water and thoroughly homogenized by shaking, resulting in fine droplets evenly distributed within the aqueous phase. The EOA was then uniformly sprayed onto the feed at a dosage of 2.0 g/kg using a high-pressure sprayer. This process was carried out daily, immediately before feeding. The fish were fed their corresponding diets once daily (7:00 AM), with the daily feed amount set at 1% to 3% of the fish’s body weight, adjusted as needed based on actual conditions. The daily management and environmental parameters during the feeding trial were consistent with those maintained during the acclimation period.

Following 8 weeks of culturing, the fish were randomly selected from each tank to collect their livers after 24 h of fasting. The livers of four fish were sampled from each tank to analyze the transcriptomes, metabolomes, histopathology, antioxidant capacity, and inflammatory indicators, respectively. All samples were immediately preserved using liquid nitrogen.

### 2.2. Histopathology Analysis

The livers were fixed in 10% formaldehyde, rinsed with 4 °C saline, and dehydrated with ethanol (70–100%). After xylene clarification and paraffin embedding, they were cut into 5–6 µm for H&E staining. The stained sections were examined and imaged.

### 2.3. Analysis of Antioxidant Capacity and Inflammatory Indicators 

The livers were homogenized in nine volumes of 4 °C sterile saline and centrifuged at 6000× *g* for 10 min. Using the ELISA method, the levels of IL-1, IL-10, and TNF-α were determined. The assay used a microplate pre-coated with antibodies specific to these cytokines. The superoxide dismutase (SOD) activity was quantified via the xanthine oxidase method, detected at 450 nm of absorbance. Catalase (CAT) activity was defined by the enzyme amount necessary to decompose 1 μmol of H_2_O_2_ per second per mg of protein. Glutathione (GSH) levels were colorimetrically determined through a reaction with 5,5′-dithiobis, forming yellow compounds with mercapto groups. The concentration of thiobarbituric acid reactive substances (TBARSs) was assessed using a thiobarbituric acid assay. A liver supernatant with a thiobarbituric acid working solution was incubated at 95 °C for 60 min to produce a red compound. The absorbance was then measured at a wavelength of 532 nm. These measurements were made with a Bio-Rad microplate reader. Data normality and the homogeneity of variances were tested and confirmed using the Shapiro-Wilk and Levene tests, respectively. Data were analyzed using Student’s *t*-test, considering *p* < 0.05 as statistically significant.

### 2.4. Transcriptome Analysis

The total RNA was isolated, and any genomic DNA contamination was eliminated by DNase I. A sequencing library was prepared from 1 μg of this RNA. The libraries were quantified using TBS380 and sequenced on an Illumina HiSeq platform with a paired-end 150 bp read. Raw reads were processed and cleaned using the Trimmomatic software (version 0.39) and then aligned to the *O. niloticus* genome using the TopHat software (version 2.1.1). Differentially expressed genes (DEGs) in the EOA vs. CON groups were identified, and transcript levels were measured using the FPKM method. The Cuffdiff software (version 2.2.1) facilitated the differential expression analysis, selecting DEGs based on criteria of a logarithmic fold change greater than 2 and an FDR of <0.05. Finally, the functional roles and pathway involvement of these DEGs were explored through a GO functional and KEGG pathway enrichment analysis.

### 2.5. Analysis of Hepatobiliary Disease Key Target Genes and Molecular Docking of EOA

Using ‘hepatobiliary disease’ as the keyword, data on hepatobiliary disease targets were obtained, screened, and organized from the GeneCards, OMIM, and TTD databases. Then, the intersection of the target genes of hepatobiliary disease and the DEGs obtained from the transcriptome analysis were considered the key target genes of hepatobiliary disease. These genes were explored in a KEGG pathway enrichment and GO functional analysis. In order to analyze and map the interaction of key targets of hepatobiliary disease in tilapia, a network of protein–protein interactions (PPIs) was constructed using the STRING database, and the organism was defined as “*Oreochromis niloticus*”. Furthermore, we used the Network Analyzer Cytoscape 3.7.2 to analyze the PPI network’s topology and rank targets based on node connectivity (degree).

Recently, AlphaFold has emerged as a prominent artificial intelligence deep-learning method for predicting protein structures. This approach has been used to simulate approximately one million structures, which are now accessible in its database. Therefore, the protein structures of the top five targets with the highest degrees in the PPI network were obtained from the AlphaFold Protein Structure Database. These structures were used to simulate the binding interactions between the main components of EOA and the receptors through molecular docking simulations using the AutoDock software (version 15.7.6). A semi-empirical free energy force field and an estimate of the conformational entropy lost during binding were employed to determine binding affinity. Prior to docking, polar hydrogens were added, and charges were assigned. Each docking calculation was performed 50 times, and the lowest energy structure was conducted using PyMOL (v2.3.0) and LigPlus (v2.2.0) for visualization.

### 2.6. Metabolomic Analysis

The liver samples were initially cooled on ice and then centrifuged at 15,000× *g* for 20 min. The supernatant was collected and diluted with LC-MS-grade water to achieve a 53% methanol concentration and was transferred to fresh tubes and centrifuged again under the same conditions. A UHPLC-MS/MS analysis was carried out using a Thermo Fisher Vanquish UHPLC system, coupled with an Orbitrap Q ExactiveTM HF mass spectrometer. Compound Discoverer 3.1 (ThermoFisher, Waltham, MA, USA) was used to determine the peak alignment, peak picking, and metabolite quantification of the raw data obtained from the UHPLC-MS/MS analysis. The identification and quantification were performed using the mzCloud, mzVault, and MassList databases. Metabolites were annotated with reference to the KEGG, HMDB, and LIPIDMaps databases.

Data normalization and OPLS-DA were conducted by the MetaboAnalystR package (version 4.0) in R. The normalization function in the MetaboAnalystR package was employed to approximate normal distributions. A univariate t-test was applied to determine statistical significance, with metabolites considered differentially significant (differentially significant metabolites, DMs) if *p* < 0.05 and with a log2 (Fold Change) of >1. Clustering heat maps were created using z-score-normalized data and the heatmap tool in tbtools. DMs were selected for an Over Representation Analysis of the KEGG enrichment analysis. The interactions between metabolites and differentially expressed genes were visualized using the MetScape plugin in the Cytoscape software (version 3.10.1).

## 3. Results

### 3.1. Liver Histopathology Alterations by Dietary EOA

**Figure 1:**
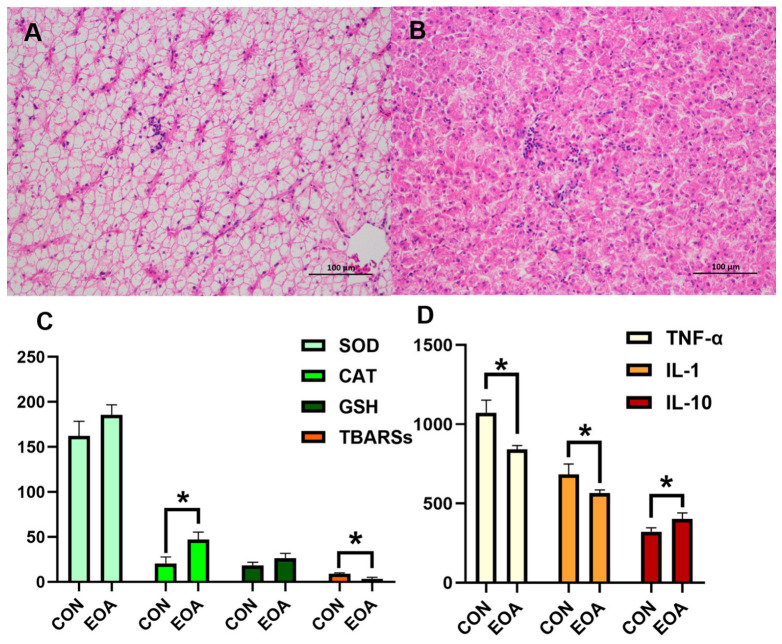
Effects of dietary EOA on the liver histological structure (HE staining, ×200) and physiological indicators of tilapia. (**A**) Liver histological structure in CON, (**B**) liver histological structure in EOA, (**C**) antioxidant parameters, (**D**) inflammatory factors. * *p* < 0.05.

In the control group, hepatocytes exhibited irregular shapes, exacerbated nuclear shrinkage and degeneration, increased vacuolization, and rendered the hepatic sinusoids unclear. In the EOA group, the liver parenchyma displayed clear and orderly hepatic plate structures, homogeneous cytoplasms, and a normal hepatic sinusoidal morphology, and the hepatocyte nuclei presented regular circular shapes located centrally within the cells (Figure 1A,B).

### 3.2. Analysis of Antioxidant Capacity and Inflammatory Indicators 

In comparison to the CON group, the CAT levels were significantly higher, and the TBARSs were significantly lower in the EOA group (*p* < 0.05, Figure 1C). Additionally, there was a significant reduction in the IL-10 and TNF-α levels and a significant increase in the IL-10 levels in the EOA group (*p* < 0.05, Figure 1D).

### 3.3. Transcriptome Analysis

Transcriptome sequencing of the liver tissues of the tilapia yielded a total of 53.41 Gb of raw data, which was filtered to obtain 50.64 Gb of clean data. An analysis of the base quality and composition revealed that the proportion of bases with a Q20 value was not less than 97.14% across all samples, and the proportion with a Q30 value was not less than 91.80%. Additionally, the GC content ranged from 42.89% to 49.27% (Table S3). These results indicate the reliability of the data, with an overall high sequencing quality, suitable for a subsequent analysis.

**Figure 2:**
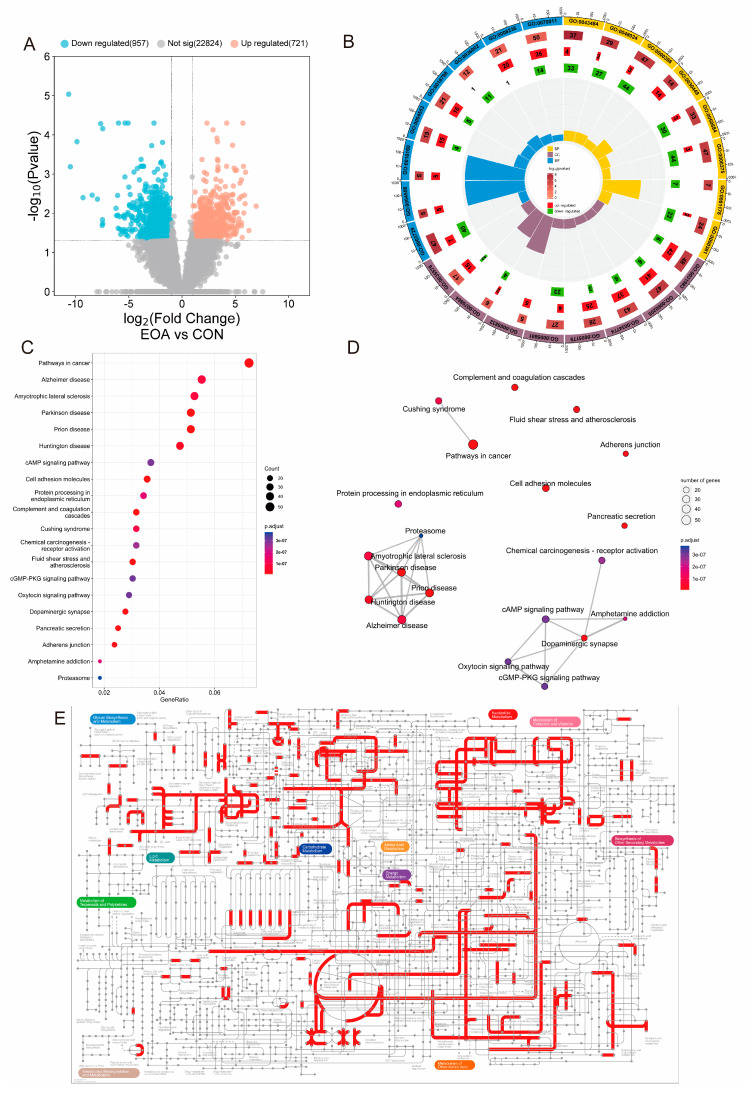
Effects of dietary EOA on the transcriptomes in tilapia livers. (**A**) Volcano plot of DEGs. (**B**) GO functional analysis of DEGs. (**C**) KEGG enrichment analysis of DEGs. (**D**) Connectivity between KEGG pathways. (**E**) Interactive pathway analysis of DEGs in livers of tilapia after dietary EOA. Red lines indicate the enriched metabolic pathways.

In the EOA versus CON group (EOA vs. CON), a total of 1068 DEGs were identified, with 721 DEGs being upregulated and 957 DEGs being downregulated (Figure 2A). The biological significance of these DEGs was then analyzed using a GO functional and KEGG enrichment analysis. In biological processes, there is a significant enrichment primarily associated with RNA expressions and splicing activities (GO0043484, regulation of RNA splicing; GO0048024, regulation of mRNA splicing; GO0000398, mRNA splicing via spliceosome; GO0050684, regulation of mRNA processing; GO0000375, RNA splicing via transesterification reactions; GO0001178, regulation of transcriptional start site selections at the RNA polymerase II promoter; GO0000381, regulation of alternative mRNA splicing via spliceosome). Regarding cellular components, there is a prominent enrichment in cell lumen components (GO0031983, vesicle lumen; GO0060205, cytoplasmic vesicle lumen; GO0034774, secretory granule lumen; GO0005775, vacuolar lumen). In terms of molecular functions, the enrichment is mainly observed in the functions of amylases, hydrolases, and peptidases (GO0003729, alpha-amylase activity; GO0004556, amylase activity; GO0016160, hydrolase activity, hydrolyzing O-glycosyl compounds; GO0004553, hydrolase activity, acting on glycosyl bonds; GO0036002, serine-type peptidase activity; GO0008236, peptidase activity, acting on L-amino acid peptides (Figure 2B, Table S4)). We further visualized expressions of genes in GO0004553, hydrolase activity, and found that genes associated with hydrolase activity, acting on glycosyl bonds, were significantly upregulated in the EOA group (Figure S1). The KEGG pathway enrichment analysis indicated that in EOA versus CON, DEGs were mainly enriched in the cancer pathway, Alzheimer’s disease, Amyotrophic lateral sclerosis, Parkinson’s disease, and prion disease (Figure 2C,D). In addition, the pathway analysis showed that some other enriched pathways in these comparisons were tightly related to carbohydrate metabolism, cofactor and vitamin metabolism, and amino acid metabolism (Figure 2E).

### 3.4. Analysis of Hepatobiliary Disease Key Target Genes and Molecular Docking of EOA

**Figure 3:**
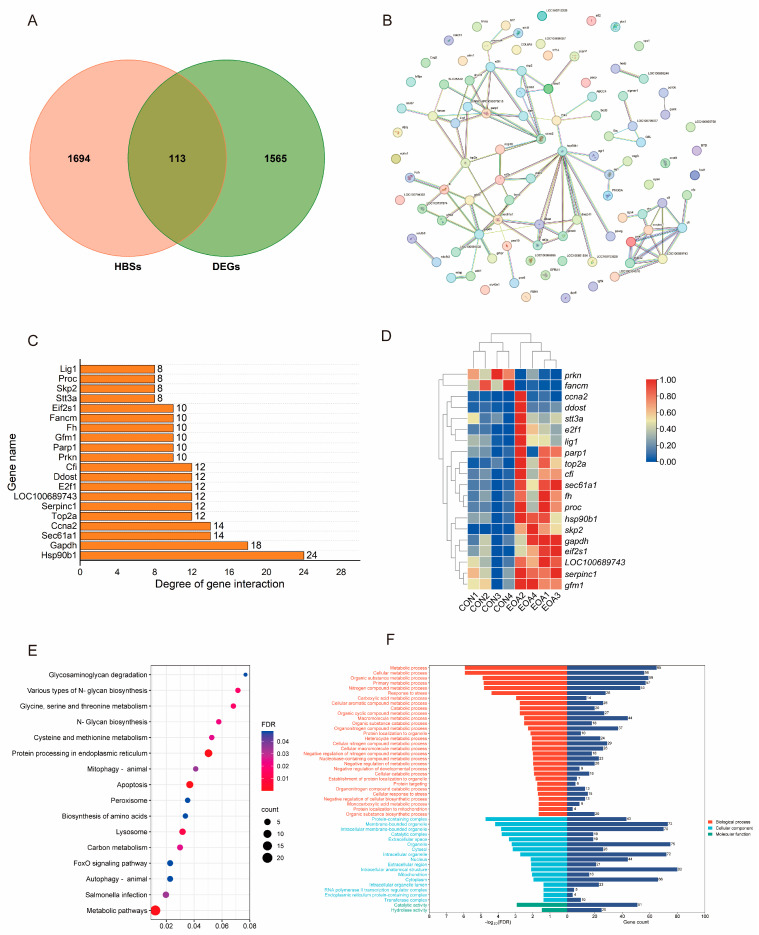
Analysis of the direct effects of EOA on hepatobiliary disease targets. (**A**) Venn plot of DEGs in transcriptome and hepatobiliary disease targets obtained from disease databases; HBSs, hepatobiliary disease targets; DEGs, differentially expressed genes. (**B**) Results of a PPI analysis using 113 EOA direct-effecting target proteins. (**C**) Degree node connectivity of the top 20 targets in the PPI network. Higher degrees indicate that these targets interact more strongly with other targets. (**D**) Gene expression heatmap of the top 20 degree node connectivity. (**E**) Results of a KEGG enrichment analysis of 113 EOA direct-effecting target genes. (**F**) Results of a GO functional analysis of 113 EOA direct-effecting target genes.

The GeneCards database obtained 1724 hepatobiliary disease targets, and the OMIM database obtained 144 hepatobiliary disease targets. The duplicate targets were removed, and 1807 targets were obtained. The intersection of DEGs and hepatobiliary disease targets included 113 targets, defined as EOA-direct effecting targets (Figure 3A). An interaction network of a total of 113 EOA direct-effecting target proteins were obtained (Figure 3B). The file was imported into the Cytoscape 3.7.2 software, and a bar chart was then generated based on its connective degree. Hsp90b1, Gapdh, Sec61a1, Ccna2, and Top2a constituted the top 5 targets based on the connective degree (Figure 3C). The 113 obtained EOA direct-effecting targets were imported into the string database for a GO and KEGG functional analysis. Moreover, the expression of genes ranking in the top 20 in terms of node connection degrees were upregulated in the EOA group (Figure 3D). These targets mainly affected the metabolic pathways and the protein processing in the endoplasmic reticulum in the KEGG pathway analysis (Figure 3E). Notably, two terms, catalytic activity and hydrolase activity, were enriched for molecular function (Figure 3F).

**Figure 4:**
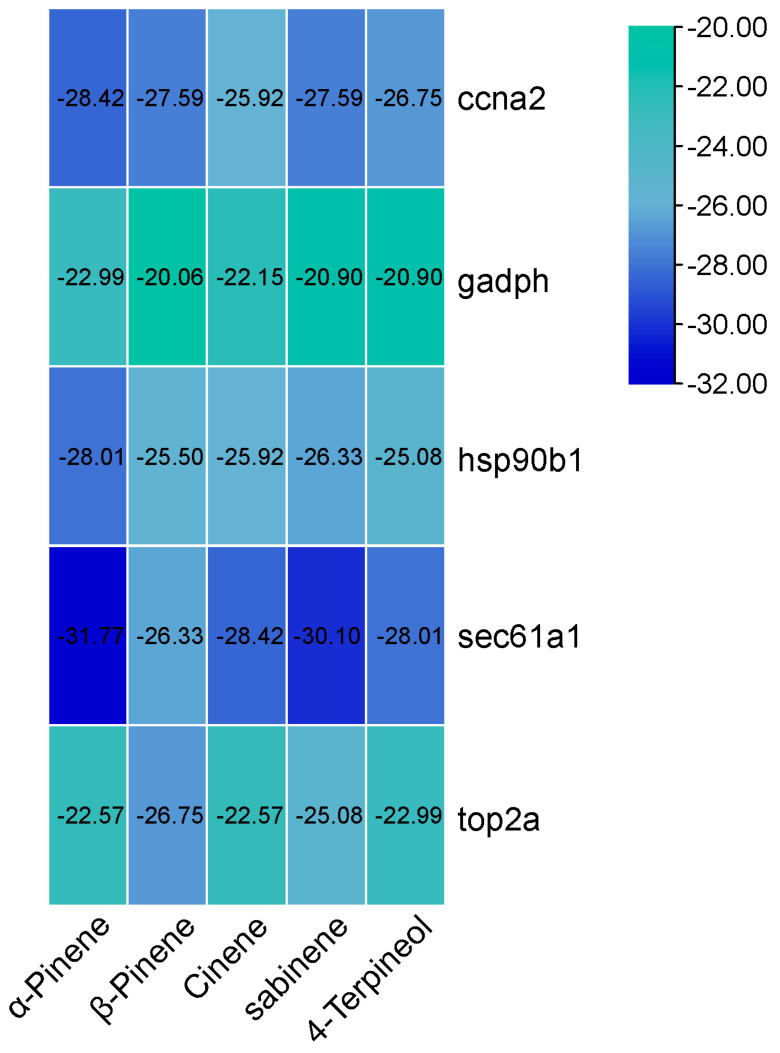
Molecular docking scoring heatmap of EOA active ingredients and top 5 targets (binding energy/kJ·mol^−1^).

**Figure 5:**
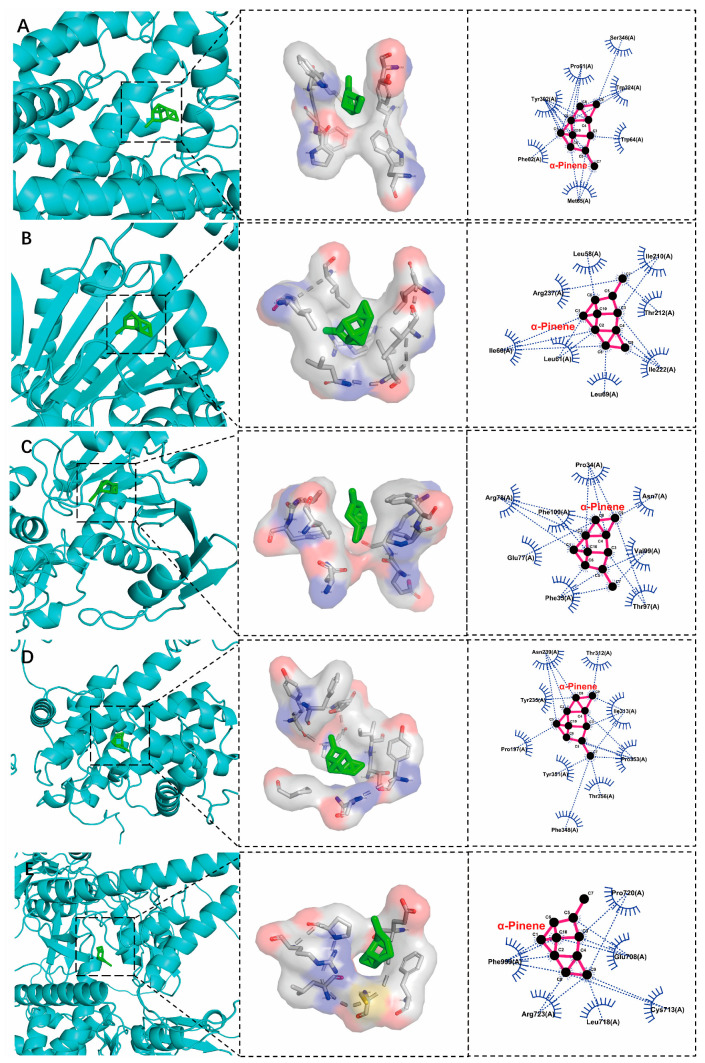
The conformational interoperability mode for docking α-pinene with targets. (**A**) Sec61a. (**B**) Hsp90b1. (**C**) Gapdh. (**D**) Ccna2. (**E**) Top2a; from left to right is the overall 3D, partial 3D, and partial 2D constructure, respectively. In 2D interactions, dotted green lines denote a hydrophobic force.

The results show that the top 5 degree targets in the PPI analysis were selected and docked with the main components of EOA using the Autodock 1.5.7 software. Among these, 25 combinations of components and targets were successfully docked, and all the docking energy was negative (Figure 4). Five representative docking models with the lowest binding energy were visualized, indicating that α-pinene accessed the active pockets of proteins. The primary binding modes between α-pinene and the proteins involved hydrophobic interactions (Figure 5).

### 3.5. Metabolomic Alterations by Dietary EOA

**Figure 6:**
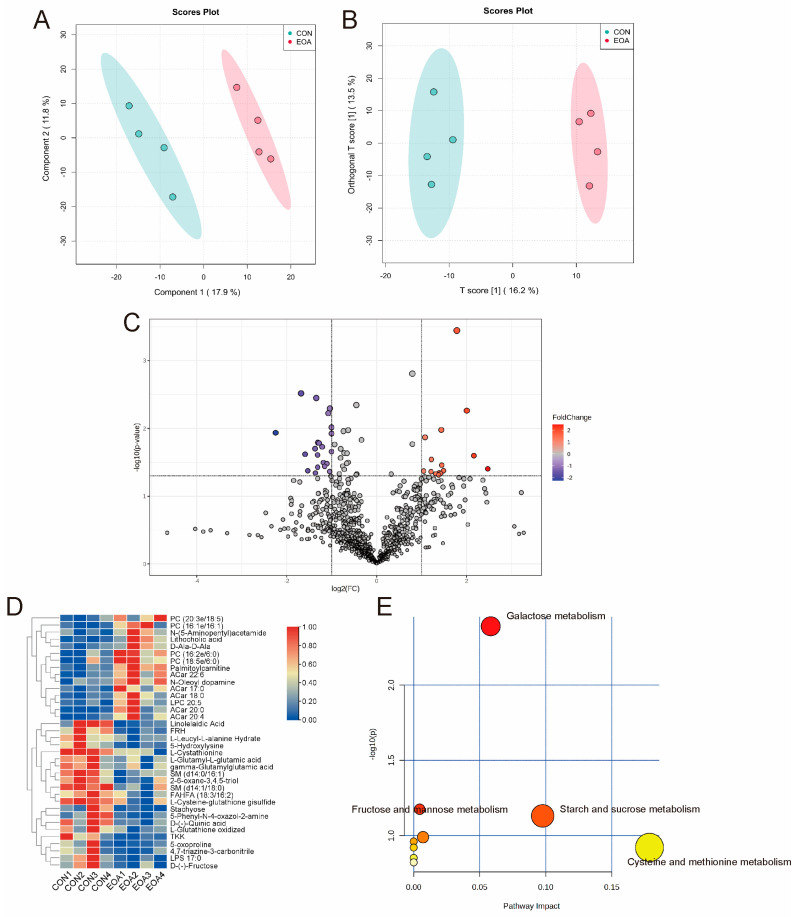
Metabolomic alterations by dietary EOA in the livers of *O. niloticus*. (**A**) PLS-DA score plot. (**B**) OPLS-DA score plot. (**C**) Volcano plot of DMs. (**D**) Heatmap of DMs. (**E**) KEGG pathway analysis of DMs.

A metabolomic analysis was used to explore the alterations in metabolic profiles resulting from dietary EOA in the livers of *O. niloticus*. The OPLS-DA showed significant differences between the two groups (Figure 6), indicating that the dietary EOA induced alterations in the metabolic phenotype of *O. niloticus* livers. Using an MS/MS analysis, a total of 1064 metabolites were identified. The predominant category was “Lipids,” accounting for 63.56%, followed by “Organic acids” (10.55%) and “Carbohydrates” (8.35%).

**Figure 7:**
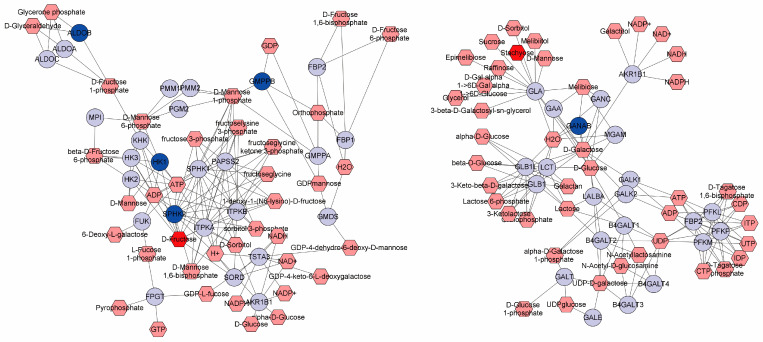
Visualization and interpretation of metabolisms of sugars and expressions of relevant genes.

Following the MS/MS analysis, we identified differential metabolites (DMs) between the CON and the EOA groups. Among these, in the EOA group, there was an increase of 16 metabolites and a decrease of 21 (Figure 6C). Among these, several phospholipids—PC (16:3e/18:5), PC (16:1e/16:1), PC (16:2e/6:0), PC (18:5e/6:0), several acylcarnitines, Acar 17:0, Acar 18:0, Acar 20:0, and Acar 20:4—exhibited significant upregulations in the EOA group, whereas saccharide substances, stachyose and D-fructose, showed significant downregulations in the EOA group (Figure 6D). A KEGG annotation analysis was performed on all DMs to identify the potential metabolic pathways affected by EOA. The most significant pathways were Galactose, Starch, and sucrose, fructose and mannose, and Cysteine and methionine metabolisms (Figure 6D). Additionally, *aldob*, *gmppb*, *hk1*, *sphk2*, and ganab were closely related to the metabolism of stachyose and D-fructose (Figure 7).

## 4. Discussion

The liver, central to homeostatic regulation through its metabolic and detoxification capacities, was the focus of our study, which assessed the implications of a diet of EOA on hepatic structure and function of tilapia. Notable effects were identified, such as an increased cellular vacuolation and a downregulation in antioxidant levels (CAT and TBARSs) and inflammatory indices (IL-1 and TNF-α) between the EOA and control group. This reflects elevated levels of inflammation in the liver and is similar to the effect of high soybean diets on fish [16,17,18]. Antioxidant enzymes are crucial for maintaining intracellular redox balance in vertebrates, helping to mitigate oxidative stress [19,20,21]. SOD and CAT are key enzymes in the antioxidant defense system [20,21]. We observed that dietary supplementation with EOA resulted in an increased activity of CAT and a reduction in the concentration of TBARSs. This suggests that EOA enhances the antioxidant defense system, thereby reducing oxidative damage. The lower TBARS levels indicate a reduction in lipid peroxidation, implying less oxidative damage to cell membranes. The inflammatory factors IL1 and TNF and the anti-inflammatory factor IL-10 were downregulated and upregulated, respectively. This suggests that an incorporation of EOA into the diet of tilapia for a period of 56 days significantly alleviated inflammatory symptoms. This is consistent with the findings observed in another study when feeding EOA to rats on a high-fat diet [22].

Using a transcriptome analysis, we found that EOA feeding altered the gene expression profiles in tilapia livers. The GO functional analysis of these DEGs revealed that the activities involved in RNA splicing, amylase and hydrolase, were the most significant terms. RNA splicing factors play roles in mRNA nuclear exports, nonsense-mediated mRNA decay, and mRNA translations. RNA splicing can produce multiple distinct mRNA transcripts from a single gene, thereby increasing the diversity of mRNAs and proteins [23]. This mechanism is crucial for the development of eukaryotic organisms and their response to environmental stress [24]. We found a significant enrichment of entries related to RNA splicing, indicating that EOA feeding altered the genetic pattern of tilapia, which in turn affected the liver function. Moreover, the related molecular functions of amylase and hydrolase were also significantly enriched. It was further found that the genes responsible for these molecular functions were significantly upregulated in the EOA group, suggesting that EOA changed the digestive ability and metabolic ability of the liver. A series disease-related KEGG pathways were significantly enriched, suggesting that EOA could significantly improve the incidence of liver and biliary diseases.

To further analyze the key role genes of EOA, we retrieved targets associated with hepatobiliary diseases and compared them to identify the subset of genes that overlap with DEGs as direct targets of EOA. Among the top 20 targets in the previous PPI network in terms of interaction strength, most are highly expressed in the EOA group. We found that many KEGG pathways are closely involved in the normal functions of the liver. Additionally, in the molecular function category of the GO functional analysis, catalytic activity and hydrolase activity were the only enriched items. Combined with the analysis of physiological indicators, it appears that EOA directly alters liver metabolic functions, increasing the metabolic intensity of the liver. Furthermore, molecular docking methods were used for targets located at the core of the PPI action network—Hsp90b1, Gapdh, Sec61a1, Ccna2, and Top2a—with the top 5 components of EOA. Our findings indicate that these small molecular compounds effectively dock with their targets, with the binding energies all being below −20 kJ/mol. Research has shown that docking binding energies of less than −5 kJ/mol are indicative of a compound ability to stably bind with proteins [25]. At appropriate concentrations, these components exhibit effects such as lowering blood sugar levels, reducing blood lipid levels, and inflammatory actions [26,27]. Interestingly, the results show that α-pinene has the highest binding strength with all targets, even though its concentration in EOA is lower than that of β-pinene. α-pinene is the main secondary metabolite in many essential oils [28]. It has been shown to have a variety of biological activities, such as antioxidant, anti-tumor, and anti-inflammatory activities [22,29]. For instance, the addition of α-pinene can significantly enhance the total antioxidant capacity of cells and can reduce the levels of oxidation [30]. Additionally, studies have shown that administering α-pinene to diabetic mice at a concentration of 0.5 mL/kg can significantly lower their blood sugar and inflammation levels [31]. Hsp90b1 is a member of the Heat Shock Protein 90 family, which plays an important role in signal transduction, the folding and degradation of proteins, and morphological evolution. Therapeutic approaches targeting Hsp90 have emerged as a potential strategy for addressing cancers and diseases linked to inflammation, with the inhibition of Hsp90 demonstrating promising outcomes in both preclinical studies and clinical trials across various cancer types and inflammatory conditions [32]. We discovered that α-pinene can bind to the active site pocket of Hsp90b1 in a temperature-dependent fashion, which might suggest that α-pinene is a potential inhibitor of Hsp90, thereby preventing the occurrence of inflammation.

A liver metabolomic analysis is a practical approach to understanding liver health [33,34]. Phosphatidylcholine (PC) is a fundamental component of lipoproteins, whose molecules are composed of fatty acids of varying lengths and degrees of saturation, playing a pivotal role in maintaining membrane structure and facilitating cellular signal transduction processes [35]. Moreover, PC possesses antioxidant properties and contributes to liver protection, such as by resisting attacks by free radicals on cell membranes and preserving the potential of mitochondrial membranes, thereby promoting the metabolism of hepatic lipid deposits [36,37]. In numerous studies, PC has also demonstrated a resistance to environmental stress [38,39,40]. The primary function of the carnitine (Acar) metabolism is to transport fatty acids into the mitochondria for fatty acid oxidation. In addition to participating in fatty acid oxidation, acetylcarnitine can also provide acetyl groups for the acetylation of proteins [41,42]. In the present study, the PC and Acar levels in the EOA group were significantly elevated, indicating EOA’s functionality in maintaining liver health. Furthermore, we discovered that the levels of stachyose and D-fructose were reduced, and pathways related to Galactose metabolism and fructose and mannose metabolism were significantly enriched. And *aldob*, *gmppb*, *hk1*, *sphk2*, and *ganab* participate in their metabolic activities. The liver stores glucose for the body and helps maintain stable and continuous levels of blood glucose and other energy substances in circulation. Depending on the body’s needs, the liver can both store and produce glucose. Coupled with the notable upregulation of genes associated with hydrolysis, we infer that EOA also enhances the function of carbohydrate metabolism in the liver.

Tilapia is considered a low-value, farmed fish in China, and the emphasis on cost reduction and economic efficiency in China’s tilapia farming model, particularly in high-density farming environments, often leads to metabolic diseases in the fish. Therefore, functional additives like EOA are becoming increasingly important in aquaculture. In this study, we selected *A. villosum* stem oil, derived from agricultural waste, which is extremely cost-effective. Therefore, the low cost and easy availability of EOA make it a promising green and efficient functional additive for aquaculture.

## 5. Conclusions

In summary, tilapia fed with EOA exhibited a healthy hepatic histology, an enhanced antioxidant capacity, and reduced inflammation levels, indicating EOA’s beneficial effects on the maintenance of liver health. Integrative transcriptomics and disease databases identified the effective components and key targets of EOA’s action. The hepatic metabolomics revealed changes in the liver phospholipid metabolism, fatty acid metabolism, and carbohydrate metabolism induced by EOA. Based on gene regulations, alterations in metabolite levels, and an analysis of liver physiological indices, it was confirmed that EOA can effectively alleviate carbohydrate and lipid metabolism disorders in the livers of tilapia, thereby promoting liver health. This knowledge will be valuable for the aquaculture industry to improve the health and welfare of tilapia in intensive farming.

## Data Availability

The authors confirm that all the data in the manuscript are available for publication.

## Supplementary Materials

The following supporting information can be downloaded at: https://www.mdpi.com/article/10.3390/antiox13091118/s1, Figure S1: Heatmap of genes expression in GO0004553: hydrolase activity; Table S1: Composition and nutrient levels of the basal diet (air-fry basis %); Table S2: The main chemical composition and content of EOA; Table S3: Quality control of transcriptome data; Table S4: Detailed information of GO functional analysis.
