# Supplementary material for: The Liver-Protective Effects of the Essential Oil from Amomum villosum in Tilapia (Oreochromis niloticus): Antioxidant, Transcriptomic, and Metabolomic Modulations
Source: Antioxidants (Basel). 2024 Sep 16;13(9):1118. doi: 10.3390/antiox13091118 (PMC11428501; doi:10.3390/antiox13091118)
Supplement: Supplementary file 1 [file antioxidants-13-01118-s001.zip › antioxidants-3171316-supplementary.pdf]

Supplementary materials

The Liver-Protective Effects of the Essential Oil from *Amomum villosum* in Tilapia (*Oreochromis niloticus*): Antioxidant, Transcriptomic, and Metabolomic Modulations

Hongbiao Dong [1,2,†], Xiangbing Zeng [1,3,†], Xiaoting Zheng [1,2], Chenghui Li [1,4], Junchao Ming [1,2] and Jiasong Zhang [1,2,*]

[1] South China Sea Fisheries Research Institute, Chinese Academy of Fishery Sciences, Key Laboratory of South China Sea Fishery Resources Exploitation & Utilization, Ministry of Agriculture and Rural Affairs, Guangzhou 510300, China; donghongbiao@163.com (H.D.); xiangbing1998@163.com (X.Z.); xtzheng1990@163.com (X.Z.); lichenghui1998@126.com (C.L.); mjc0312@126.com (J.M.)

[2] Key Laboratory of Efficient Utilization and Processing of Marine Fishery Resources of Hainan Province, Lingshui 572426, China

[3] Key Laboratory of Mariculture, Ministry of Education, Ocean University of China, Qingdao 266003, China

[4] National Aquaculture Engineering Technology Research Center, Zhejiang Ocean University,
Zhoushan 316000, China

* Correspondence: jiasongzhang@hotmail.com

† These authors contributed equally to this work and should be considered co-first authors.

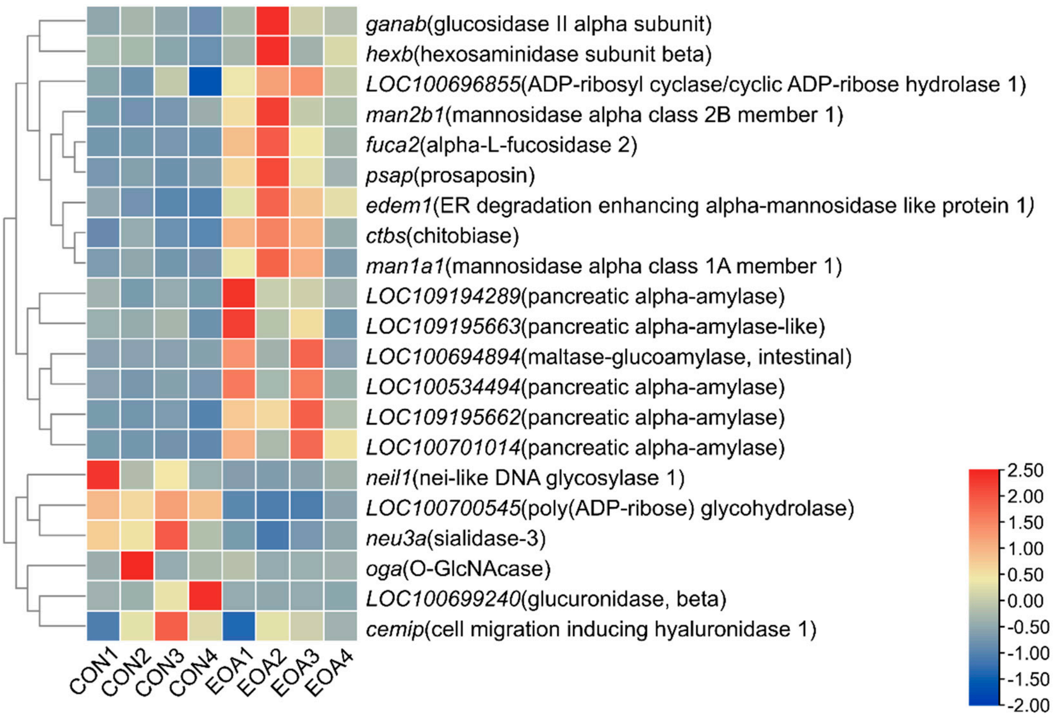

Figure S1. Heatmap of genes expression in GO0004553: hydrolase activity

Table S1

Composition and nutrient levels of the basal diet (air-fry basis %).

Ingredients	Content	Nutrient levels	Content
Soybean meal	25	Crude protein	30.90
Rapeseed meal	12	Crude fatty	5.59
Soy protein concentrate	9.5	Ash	12.04
Soybean oil	2.10	Moisture	10.54
Rice bran	29.5		
Flour	17.50		
Choline chloride	0.05		
Vitamin and mineral premix	2.00		
Carboxymethylcellulose sodium	1.00		
Diatomite	1.35		
Total	100		

Vitamin and mineral premix provided the following per kg of the diet Vitamin D3 1 000 000 IU, Vitamin A3 200 000 IU, Vitamin E 0.016 g, Ca 10 500 mg, Se 100 mg, Mg 5 500 mg, Cu 8 000 mg, Zn 10 500 mg, Mn 550 mg.

Table S2

The main chemical composition and content of EOA.

Number	Name	Precent（%）
1	β-Pinene	44.67
2	α-Pinene	29.16
3	Sabinene	14.43
4	Cinene	1.974
5	4-Terpineol	1.179
6	4-Cymene	1.012
7	β-Myrcene	1.010
8	β-Phellandrene	0.677
9	γ-Terpinene	0.606
10	Cineole	0.330
11	Myrtenal	0.327
12	Edulan	0.250
13	Camphene	0.214
14	Pinocamphone	0.214
15	Caryophyllene	0.214
16	Isocarvone	0.195
17	α-Terpinene	0.191
18	Isoterpinolene	0.189
19	Carvone	0.175
20	Myrtenol	0.156
21	β-Ocimene	0.130
22	α-Fenchene	0.081
23	β-Elemene	0.079
24	α-Caryophyllene	0.077
25	Para-cymene-8-ol	0.041

Table S3

Quality control of transcriptome data.

Sample id	Total reads	Total bases	Q20 rate	Q30 rate	Read1 mean length	Read2 mean length	GC content
CON1	41529078	5951496857	0.972141	0.91801	143	143	0.440541
CON2	49695768	7129615813	0.97144	0.919185	143	143	0.438223
CON3	44578936	6385615216	0.9777	0.931836	143	143	0.436648
CON4	43954010	6288424643	0.980257	0.938013	143	142	0.428912
EOA1	43185308	6216524206	0.982715	0.943751	144	143	0.492735
EOA2	43353228	6202141183	0.983513	0.946275	143	143	0.476537
EOA3	45207646	6439288750	0.983592	0.946739	142	142	0.486959
EOA4	43066218	6016934926	0.980949	0.940001	139	139	0.45293

Table S4

Detailed information of GO functional analysis.

GO term	Description	Category	*P* value
GO0043484	regulation of RNA splicing	BP	1.36E-08
GO0048024	regulation of mRNA splicing, via spliceosome	BP	2.73E-07
GO0000398	mRNA splicing, via spliceosome	BP	3.21E-07
GO0030449	regulation of complement activation	BP	4.18E-07
GO0050684	regulation of mRNA processing	BP	4.60E-07
GO0000375	RNA splicing, via transesterification reactions	BP	4.90E-07
GO0001178	regulation of transcriptional start site selection at RNA polymerase II promoter	BP	9.11E-07
GO0000381	regulation of alternative mRNA splicing, via spliceosome	BP	9.66E-07
GO0031983	vesicle lumen	CC	3.28E-07
GO0060205	cytoplasmic vesicle lumen	CC	3.50E-07
GO0034774	secretory granule lumen	CC	2.84E-06
GO0005775	vacuolar lumen	CC	3.84E-06
GO0005681	spliceosomal complex	CC	9.15E-06
GO0005832	chaperonin-containing T-complex	CC	4.33E-05
GO0070864	sperm individualization complex	CC	6.57E-05
GO0035578	azurophil granule lumen	CC	7.67E-05
GO0003729	alpha-amylase activity	MF	1.22E-07
GO0004556	amylase activity	MF	9.06E-07
GO0016160	hydrolase activity, hydrolyzing O-glycosyl compounds	MF	9.06E-07
GO0004553	hydrolase activity, acting on glycosyl bonds	MF	1.72E-06
GO0016798	pre-mRNA binding	MF	7.00E-06
GO0036002	serine-type peptidase activity	MF	0.00011
GO0008236	peptidase activity, acting on L-amino acid peptides	MF	0.000195
GO0070011	aminoacyl-tRNA editing activity	MF	0.00023
